# An Underwater Acoustic Target Recognition Method Based on Restricted Boltzmann Machine

**DOI:** 10.3390/s20185399

**Published:** 2020-09-21

**Authors:** Xinwei Luo, Yulin Feng

**Affiliations:** Key Laboratory of Underwater Acoustic Signal Processing of Ministry of Education, Southeast University, Nanjing 210096, China; 220190901@seu.edu.cn

**Keywords:** restricted Boltzmann machine, GFCC, auto-encoding, underwater acoustic, ATR

## Abstract

This article focuses on an underwater acoustic target recognition method based on target radiated noise. The difficulty of underwater acoustic target recognition is mainly the extraction of effective classification features and pattern classification. Traditional feature extraction methods based on Low Frequency Analysis Recording (LOFAR), Mel-Frequency Cepstral Coefficients (MFCC), Gammatone-Frequency Cepstral Coefficients (GFCC), etc. essentially compress data according to a certain pre-set model, artificially discarding part of the information in the data, and often losing information helpful for classification. This paper presents a target recognition method based on feature auto-encoding. This method takes the normalized frequency spectrum of the signal as input, uses a restricted Boltzmann machine to perform unsupervised automatic encoding of the data, extracts the deep data structure layer by layer, and classifies the acquired features through the BP neural network. This method was tested using actual ship radiated noise database, and the results show that proposed classification system has better recognition accuracy and adaptability than the hand-crafted feature extraction based method.

## 1. Introduction

Underwater acoustic target recognition is the technique of identifying the type of target through the analysis of underwater acoustic signal. The hardware basis of underwater acoustic target recognition is sonar equipment, which converts underwater acoustic wave into digital signal through hydrophones, and obtains underwater target information through various signal processing methods [1]. Underwater acoustic target recognition has become one of the main functions of sonar systems [2]. Feature extraction and pattern recognition are the key steps of an underwater acoustic target recognition algorithm. Feature extraction is the process of obtaining features from the original signal. Commonly used algorithms include traditional time-frequency graph methods [3], auditory perception methods [4,5], and multi-dimensional feature fusion methods [6]. Pattern recognition algorithm is to divide the samples into certain categories according to the characteristics of the samples. Traditional pattern recognition algorithms include linear discriminant analysis (LDA), support vector machine (SVM) [7], Gaussian mixture model (GMM) [8], etc. In recent years, the neural network method has also been widely used in underwater acoustic signal pattern recognition [9,10,11,12].

The digital signals recorded by sonar through analog-to-digital conversion contain a lot of information about underwater acoustic targets [13,14,15,16]. What we need is information (features) that is helpful for target recognition. Feature extraction is a method and process of extracting characteristic information from a large number of data. The traditional feature extraction methods are mainly hand-crafted feature extraction [17]. These methods are based on a kind of physical or statistical characteristics of underwater acoustic target signal and select specific signal components as the feature of target recognition through signal processing.

Because of the short-time stationary characteristic of underwater acoustic signal, LOFAR spectrum is an effective representation of signal. LOFAR is a typical method of passive sonar signal processing. It is based on the short-time Fourier transform (STFT) to obtain the time-frequency spectrum of the signal and uses the time accumulation of the low-frequency line spectrum to detect the line spectrum. Each row of data in LOFAR spectrum corresponds to the original power spectrum of a frame of underwater acoustic signal data. The original power spectrum can be directly used as the input of a recognition system for pattern recognition. However, the amount of original power spectrum data is very large, which brings great pressure to the training and work of recognition system. To reduce the complexity of classification, a lot of work has been carried out to further reduce the data dimension. Common methods are KL transformation [18] and principal component analysis (PCA) [19].

Human auditory system also has a good ability to identify ship radiated noise. To simulate the human ear hearing, people have deeply studied the human ear hearing system. The processing of sound by auditory system mainly includes the decomposition of acoustic signal by cochlea of inner ear, energy conversion from sound wave to nerve by hair cell vibration, and analysis of acoustic spectrum characteristics by inhibition side network. [20]. In the processing of simulated human hearing, Mel band-pass filter bank is used to simulate the decomposition of the acoustic signal of the cochlea; the DCT transform is used to simulate the energy conversion caused by the vibration of the hair cell; and the pattern recognition method is used to complete the analysis of the acoustic spectrum characteristics. Speech recognition is ahead in the field of human ear hearing for acoustic signal recognition, forming a routine process of preprocessing, framing, MFCC/GFCC feature extraction, and Hidden Markov Model (HMM) pattern matching. Using auditory features for underwater acoustic target recognition can obtain a better recognition rate than the Autoregressive model (AR) model [21].

From the perspective of information theory, hand-crafted feature extraction is actually a process of subjective screening of original information to reduce the amount of information [22]. When the characteristics of the data are inconsistent with the subjective assumptions, the classification performance of hand-crafted features will be greatly reduced. In addition, traditional unsupervised clustering methods such as K-means and Gaussian mixture models have more limitations in practical applications. First, the number of clusters must be given when clustering, which is difficult to achieve in practical applications. Second, assuming that the sample characteristics follow a specific distribution, GMM requires that the samples conform to a Gaussian mixture distribution, but real samples are often difficult to fit with a Gaussian distribution, which will result in a distribution mismatch and reduce the clustering effect [23].

Under the noise of the marine environment, the SNR of the underwater acoustic signal is relatively low. An important flaw in the realization of underwater acoustic target recognition through hand-crafted features is that it is difficult to adaptively adjust the feature extraction scale when the SNR is low. The clustering model needs to be modified according to the actual environment to obtain good classification effect. In addition, because the actual underwater acoustic signal samples are extremely diverse and difficult to obtain, the generalization ability of the hand-crafted features is poor, and it is difficult to achieve a consistent effect on various signals [24]. Feature extraction based on various neural network methods can adaptively extract the key information of the acoustic signal according to the probabilistic characteristics of the data, and it will have a better effect in the problem of multi-class division.

The recent development of deep neural networks (DNN) has given a better method than traditional hand-crafted feature extraction for extracting deep structures of complex data. Using DNN to extract features is widely used in various fields [25]. There are many specific forms of feature extraction using DNN, such as auto-encoder method [9,26], convolutional neural network method [10], deep Boltzmann machine method (DBM) [11], etc. Among them, the Boltzmann machine is very suitable for acoustic feature extraction because of its characteristics: (1) The Boltzmann machine is a neural network that generates a probability model based on data and can be trained unsupervised. (2) DBM extracts the deep structure of the input data set and obtains the features by auto-encoding. Research shows that this feature has stronger performance than hand-crafted features [27]. The experiments presented in this paper also prove this.

The deep Boltzmann machine is an undirected graph-based depth generation algorithm proposed by Hinton [11], which is modified from the deep confidence network [28]. The basic unit of DBM is a restricted Boltzmann machine (RBM) with a two-layer bidirectional structure. This structure optimizes the network parameters by minimizing the energy function based on the probability distribution of the data. The DBM model is widely used in human voice, image recognition, and other fields [29]. DBM is composed of multi-layer RBM stack. This kind of network has the characteristics of self-supervised training, which is suitable for the scene of underwater acoustic target recognition which lacks labeled data and can supplement the generalization ability of unknown signals for traditional feature extraction methods [30].

In this paper, an auto-encoder based on Boltzmann machine is constructed to perform adaptive feature extraction on the input signal. After layer-by-layer greedily pre-training, the overall optimization is performed by Markov chain Monte Carlo. The result of feature extraction can be used to classify and recognize signals through neural network after dimensionality reduction. It can obtain recognition performance better than hand-crafted features for ordinary noise signals. Experiments show that the system is also suitable for underwater acoustic signals. The important symbols involved in the description of the algorithm principle are shown in Table 1.

The remaining content of the article is structured as follows. Section 2 introduces the detailed information and learning algorithm of the feature extraction of acoustic signal. Section 3 introduces the detailed principle of the target recognition system. Section 4 gives the experimental results based on two databases. Section 5 is the conclusion of the article.

## 2. Feature Extraction of Underwater Acoustic Signal

The generation mechanism of ship radiated noise is very complicated. The sources of these noises including propellers, rotating and reciprocating machinery, various pumps, etc. According to different noise sources, noise can be divided into several components, including mechanical noise, propeller noise, and hydrodynamics noise etc. [31].

**Figure 1:**
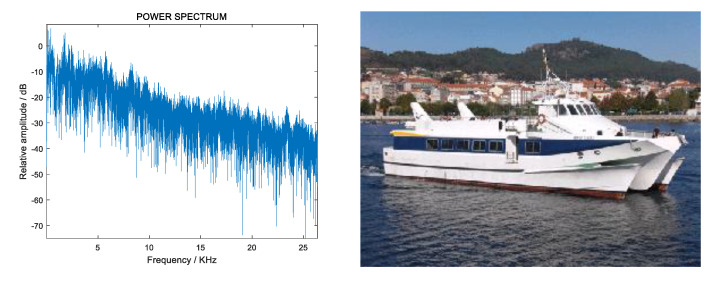
Radiated noise power spectrum (**left**) and picture (**right**) of a passenger ship.

**Figure 2:**
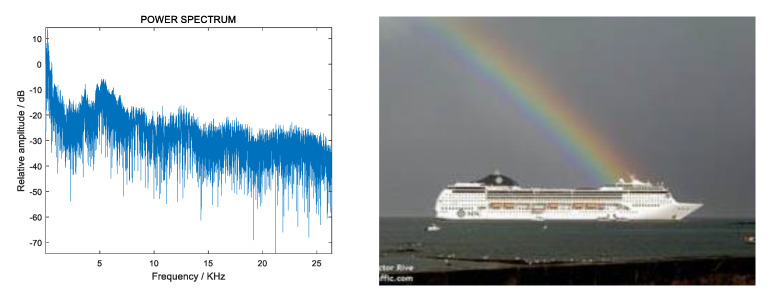
Radiated noise power spectrum (**left**) and picture (**right**) of an ocean liner.

Ship radiated noise is approximately stable in a short time, thus it can be described by power spectrum. The power spectrum of the signal contains the characteristics of the ship and reflects its sailing status. Radiated noise power spectrum and pictures of ships [32] are shown in Figure 1 and Figure 2.

Figure 1 and Figure 2 show the difference in the power spectrum of the radiation noise of different ships. Most of the features used for target recognition are constructed based on the power spectrum, including LOFAR, MFCC, GFCC, etc. However, these features are extracted based on a preset model, which is poor in robustness in practical applications.

This section mainly introduces the principles of hand-crafted underwater acoustic feature extraction methods, including GFCC and LOFAR, and proposes an auto-encoding feature extraction method based on improved deep Boltzmann machine.

### 2.1. Hand-Crafted Feature Extraction

The information removal process of hand-crafted feature extraction methods such as GFCC and LOFAR are based on the information extraction process judged by experts.

**Figure 3:**
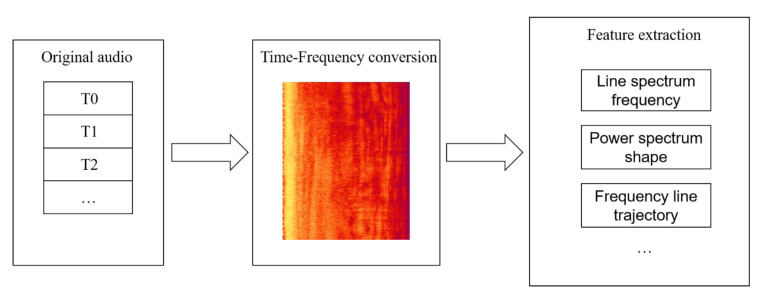
Schematic diagram of the LOFAR method.

The LOFAR spectrogram is a classic time-frequency analysis method, which is widely used in underwater acoustic target recognition. Because the noise has short-term stability characteristics, the signal is sampled by the short-window function sliding method, and the power spectrum estimation of the signal at different moments can be obtained through the short-time Fourier transform. The three-dimensional graph of time, frequency, and power can be drawn, that is, the LOFAR spectrum. The LOFAR spectrum contains two-dimensional information of time and frequency, and the information is redundant. Classification using the original LOFAR spectrum will put a lot of pressure on the recognition system. Therefore, reduction of the amount of information is necessary. Common methods are KL transformation and principal component analysis. The main features that help target recognition in LOFAR spectrogram include line spectrum frequency, power spectrum shape, and frequency line trajectory. Figure 3 describes the implementation process of the LOFAR feature extraction method.

Line spectrum information and line spectrum track are the reflection of narrow band components in target signal, which are vulnerable to multi-target interference. The shape of power spectrum is also vulnerable to the interference of environmental noise and multi-target. Therefore, it is difficult to obtain satisfactory results by using the features alone. The LOFAR spectrogram retains the original information of the underwater acoustic signal to a great extent, but it ignores sonarist’s hearing in recognition. Therefore, many scholars try to study the underwater acoustic target recognition algorithm based on auditory perception.

**Figure 4:**
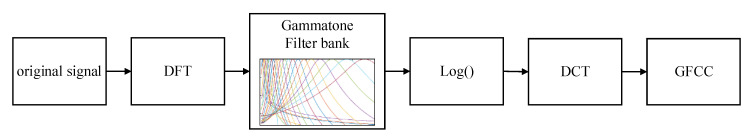
GFCC algorithm flow chart.

GFCC uses Gammatone filters to divide the acoustic signal over a period of time into different frequency band components. The Gammatone power spectrum is taken as a logarithm. Finally, the GFCC features are extracted by discrete cosine transforms (DCT). The Gammatone power spectrum is taken as a logarithm. It is a signal processing structure that imitates the human cochlea, and its performance will be close to the best sonarist’s resolution effect theoretically, but frequency components outside the hearing range will be discarded. Figure 4 describes the implementation process of the GFCC feature extraction method. The detailed implementation process of the GFCC is shown in Table 2.

The hand-crafted feature is actually an expert system. GFCC is a method based on human auditory model, which has a good theoretical basis and is easy to implement. This method has good performance for speech signal. However, the hand-crafted features subjectively discard some features that help identify the target. Therefore, GFCC method is often not effective in real marine environments.

### 2.2. Probability Model of the Deep Boltzmann Machine

**Figure 5:**
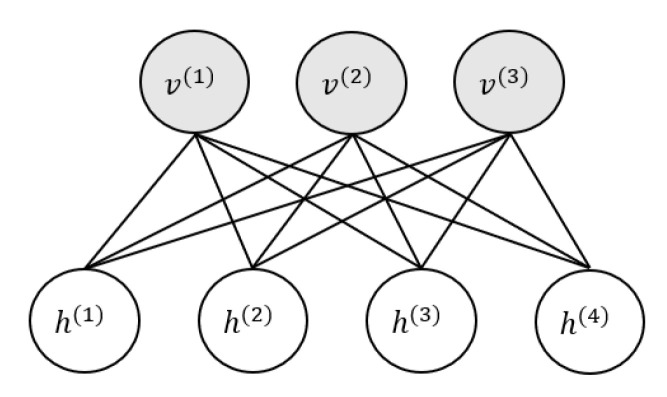
RBM structure.

Deep Boltzmann machine transforms low-level input data into high-level features through unsupervised layer-by-layer training. These high-level features represent the complex dependencies implied in the data. The deep Boltzmann machine can be decomposed into a deep neural network formed by stacking multiple restricted Boltzmann machines (RBM). Each RBM implements auto-encoding, and each auto-encoding can extract features that are more abstract. The basic structure of RBM is shown in Figure 5. It is an undirected bipartite graph. It constructs a fully connected structure between the visible layer and the hidden layer to perform auto-encoding and reconstruction.

The learning process of Boltzmann machine is to reduce the energy function of the whole system in the simulated annealing process, so that the system tends to be ideal. The energy function of the binary RBM system is:

(1) $$E\left(v,h\right)=-b^{\prime }v-c^{\prime }h-h^{\prime }Wv$$

where W is the connection weight of RBM. b and c are the offset of the visible layer and the hidden layer, respectively.

To correspond the low energy to the ideal system state, the energy-based joint probability distribution of the visible layer and the hidden layer is defined as:

(2) $$P\left(x\right)=\underset{h}{\sum }p\left(x,h\right)=\underset{h}{\sum }\frac{e^{-E\left(x,h\right)}}{Z}$$

where $Z=\underset{x}{\sum }e^{-F\left(x\right)}$ is the partition function and $F\left(x\right)=-log\underset{h}{\sum }e^{-E\left(x,h\right)}$ is the free energy.

Based on the structure of RBM, there is no connection between the units of each layer, and their distributions are independent of each other, thus the distribution of visible units and hidden units in RBM can be summarized by the following formula.

(3) $$p\left(h|v\right)=\underset{i}{\prod }p\left(h_{i}|v\right)\;p\left(v|h\right)=\underset{j}{\prod }p\left(v_{j}|h\right)\;$$

Substituting the system energy function and probability distribution function into the above formula, the simplification yields:

(4) $$p\left(h_{i}=1|v\right)=\mathrm{sigm}\left(c_{i}+W_{i}v\right)\;p\left(v_{j}=1|h\right)=\mathrm{sigm}\left(b_{j}+W_{j}^{’}h\right)$$

where sigm(x) is sigmoid function, the probability distribution of each neuron in any state can be obtained using Equation (4). Then the probability distribution of the whole system can be obtained.

Substituting Equations (1)–(4) into the random gradient, the log-likelihood gradient of the binary RBM is obtained.

(5) $$\begin{array}{l} -\frac{\partial \log p\left(v\right)}{\partial W_{ij}}=E_{v}\left[p\left(h_{i}|v\right).v_{j}\right]-v_{j}^{\left(i\right)}.sigm\left(W_{i}.v^{\left(i\right)}+c_{i}\right)\\ -\frac{\partial \log p\left(v\right)}{\partial c_{i}}=E_{v}\left[p\left(h_{i}|v\right)\right]-sigm\left(W_{i}.v^{\left(i\right)}\right)\\ -\frac{\partial \log p\left(v\right)}{\partial b_{j}}=E_{v}\left[p\left(v_{j}|h\right)\right]-v_{j}^{\left(i\right)} \end{array}$$

Gibbs sampling is usually used to reduce the computational complexity of sample estimation. The Gibbs sampler of N variables $S=\left(S_{1},\ldots ,S_{N}\right)$ is completed by N sampling subsequences in the form of $S_{i}\sim p(S_{i}|S_{-i})$, where $S_{-i}$ contains all variables in S except $S_{i}$.

**Figure 6:**
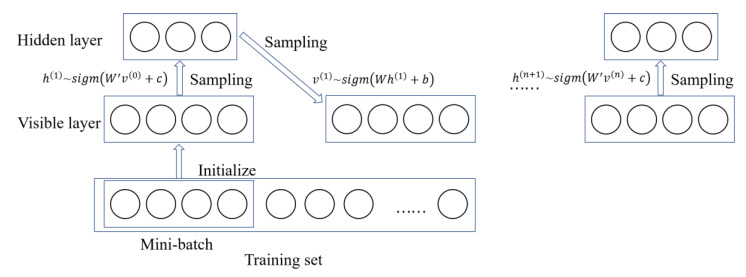
Gibbs sampling.

In RBM, S includes all visible and hidden units. Given a fixed value of hidden units, visible cells can be sampled. Similarly, hidden units are sampled when the value of the visible units is determined. Therefore, the steps in the Markov chain are as follows:

(6) $$h^{\left(n+1\right)}\sim sigm\left(W^{\prime }v^{\left(n\right)}+c\right)\;v^{\left(n+1\right)}\sim sigm\left(Wh^{\left(n+1\right)}+b\right)$$

where $h^{\left(n\right)}$ represents all hidden units in the nth step of the Markov chain and $v^{\left(n\right)}$ represents all visible units in the nth step of the Markov chain. The result of each sampling is a set of binary random variables subject to the above distribution. The schematic diagram of Gibbs sampling is shown in Figure 6. The Gibbs sampling is performed until the Markov chain converges, and the Gibbs sampling value is the estimated value expectation in Equation (5).

Although Gibbs sampling is an effective method to solve approximate expectation and update RBM weights through Markov chain Monte Carlo method, it needs continuous computation until the Markov chain is stable. The contrast divergence (CD) algorithm proposed by Hinton [33] can effectively accelerate the learning of RBM while maintaining the accuracy of the algorithm. CD algorithm measures the distance between the estimated probability distribution and the real probability distribution and takes it as the measurement criterion. Using a small part of all training samples to build mini-batch and run n-step Gibbs sampling can get the expectation of all samples. This expectation is used to update RBM parameters by stochastic gradient method:

(7) $$\Delta W\approx \varepsilon \left(E_{P_{data}}\left[vh^{T}\right]-E_{P_{n}}\left[vh^{T}\right]\right)$$

where $P_{n}$ is the probability distribution obtained by n-step Gibbs sampling. Generally, a good result can be achieved by taking n as 1.

Although CD algorithm is widely used, it is found that CD algorithm only performs well in the early training period. In the late training period, the computational efficiency of CD algorithm decreases because the ergodicity of Markov chain decreases. The persistent contrast divergence (PCD) algorithm proposed by Tieleman [34] has better RBM learning ability and improves the defect that CD algorithm cannot maximize the likelihood function. PCD algorithm parameter update rule is:

(8) $$\Delta W\approx \varepsilon \left(E\left[v{h_{0}}^{T}\right]-E\left[v_{t+k}{h_{t+k}}^{T}\right]\right)$$

where $\left(v_{t+k},\;h_{t+k}\right)$ is the sample obtained by running k steps of Gibbs sampling from $v_{t}$ starts. The difference between PCD algorithm and CD algorithm is that the new unit values in Markov chain are used for sampling. After parameter updating, only k-step Gibbs sampling is needed to extract new samples without computing new Markov chain. The advantage of this improvement is that Markov chain can better describe the current state of the model.

In the design of underwater acoustic target recognition system, the convergence speed and recognition performance of the algorithm are important, which requires the algorithm to make a trade-off between high precision and low delay. In this paper, cd-k algorithm is used to train RBM auto-encoder, and the convergence speed and performance of the model are adjusted by variable learning rate.

### 2.3. RBM Auto-Encoder

The main application of RBM in neural network algorithm is pretraining. Each layer of the network is trained by greedily training, and the parameters are copied into the neural network as initial values. Training from these initial parameters can make the network converge quickly.

The RBM described in Section 2.2 actually acts as an automatic encoder in the system. The core purpose of RBM is to compress high-dimensional raw data into low-dimensional features from the perspective of data probability distribution and provide more effective input for classification system. In this process, the data dimension is compressed, which is called an incomplete auto-encoder. The most commonly used auto-encoder is Principal Component Analysis (PCA), which uses a linear activation function to project data onto the hyperplane closest to the Euclidean distance, thereby obtaining low-dimensional features with minimal loss of information. In particular, RBM is a generation model. According to its probability model, this model can generate random data in accordance with the original data probability model.

**Figure 7:**
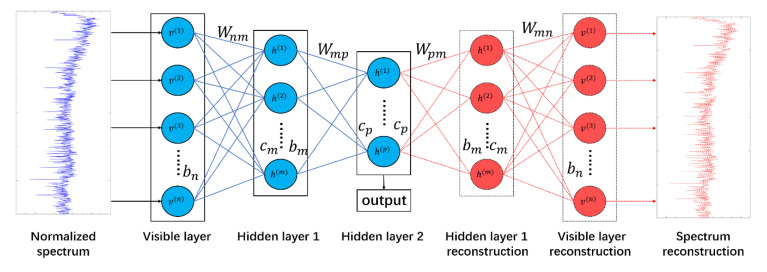
RBM auto-encoder structure.

To extract features that are more abstract, we stack the auto-encoders to obtain the auto-encoder shown in Figure 7, which is consistent with the multi-hidden layer structure of the neural network. The output of the previous layer is used as the input and self-supervised objective function at the same time, and the RBM auto-encoder is greedily trained layer by layer to obtain the final multi-layer structure. In the auto-encoder, each layer of auto-encoder is to obtain the data probability model of the previous-level encoder to accurately reconstruct it. This structure can significantly reduce the computational complexity of abstract feature extraction.

The multi-layer RBM used in the RBM feature extraction system in this paper is actually a stack-type auto-encoder. The two-layer structure of each layer of RBM takes the previous layer as an input, obtains its probability model, and uses the Gibbs sampler for greedily training to reach the final refactor accurately.

When RBM auto-encoder model is used in actual signal processing, a noticeable problem is that the signal needs to be matched with the model through preprocessing. The energy of underwater acoustic signal is mainly concentrated in low frequency, and high frequency signal is often submerged in background and interference signal. Through the observation of data, the first 8 kHz of signal is used in underwater acoustic signal processing. At the same time, the underwater acoustic signal has a longer time stability than the voice signal, thus this paper adopts the data frame length of 100 ms, which is more than the tens of milliseconds in speech signal analysis, and uses 50% sliding framing.

On the other hand, the hyperparameters of the model need to be manually set to adapt to the actual underwater acoustic signal. The number of units in the visible layer should be consistent with the number of sampling points of the normalized spectrum after the effective frequency is intercepted. The number of units in each hidden layer needs to be set decrementally layer by layer on the basis of the visible layer until it reaches a low dimension. Other hyperparameters such as learning rate, minibatch size, and number of model layers need to be found through experiments to find suitable values for performance and convergence speed.

The function of the RBM auto-encoder is to extract the probability characteristics of the data distribution through layer-by-layer unsupervised training. The Boltzmann energy equation (Equation (1)) based on the undirected graph can evaluate the energy function of the current state of the network. The energy function reflects the probability of occurrence of the current network state through the probability-energy equation (Equation (2)). Conditional probability equation (Equation (4)) uses the current network parameters and input unit state to calculate the state of the hidden layer unit, and reconstructs the state of the input unit through the state of the hidden layer unit, which is the basis for the realization of the Gibbs sampler. Gibbs sampling (Equation (6)) is used to solve the Markov chain to obtain the mathematical expectation including all input and hidden layer unit required by the stochastic gradient parameter update formula (Equation (5)). The detailed implementation process of the RBM auto-encoder is shown in Algorithm 1.
**Algorithm 1** RBM Auto-encoder1: Configure the input, adjust the input dimension of RBMs and input structure2: Randomly initialize the entire system parameters, including connection weights and offsets3: Preprocess the acoustical signal get Spectrum4: for i = 1:layer5:  for j = 1:epoch[i]6:   for batch = 1:batch_num7:    Complete the forward process according to Equation (6), and calculate the hidden unit state8:    Complete the reconstruction process according to Equation (6), and calculate the state of the visible cell for reconstruction9:    Update model parameters according to Equation (5)10:   end11:  end12: end

## 3. Target Recognition System

**Figure 8:**
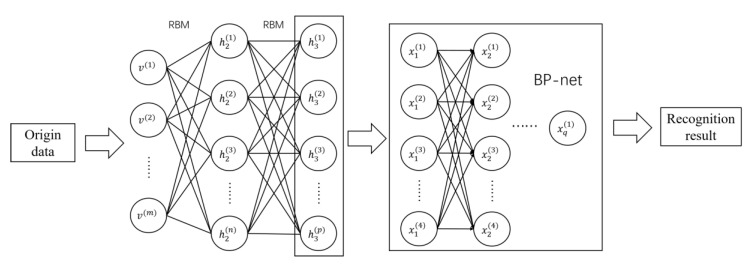
Deep clustering system structure.

The clustering model mainly includes two components, a L-layer RBM auto-encoder and a BP neural network classifier, and the output node of the Boltzmann machine is used as the input of the neural network. The structure of the entire deep clustering system is shown in Figure 8. The two subsystems are cascaded. The auto-encoder uses a CD-k sampler to perform unsupervised training layer by layer.

The input to the system is the normalized spectrum of the acoustic signal. The deep Boltzmann machine acts as an auto-encoder in the clustering system to extract deep data features from the original input. With the increase of the levels of deep Boltzmann machines, the output features become more abstract. Abstract features with certain dimensions are conducive to the subsequent neural network clustering.

BP neural network is the most commonly used neural network classifier. BP neural network with many hidden layers is also called multi-layer perceptron or deep neural network. The data flow of BP neural network always flows from the input layer through the hidden layer to the output layer.

The learning algorithm of BP neural network is the process of learning the network parameters under the supervision of data and labels. The network parameters include the connection weight matrix W and the neuron threshold θ of each layer of the network. The learning algorithm can be divided into two processes, forward propagation and back propagation. When forward propagation, the multi-layer structure uses the neuron of the previous layer as input, and calculates the value of the neuron of the current layer with the current network parameters.

(9) $$x_{j}^{\left(p+1\right)}=f\left(\overset{n-1}{\underset{i=0}{\sum }}{W_{ij}}^{\left(p\right)}x_{i}^{\left(p\right)}-{\theta_{j}}^{\left(p\right)}\right),j\in \left[0,n-1\right]$$

where $x_{i}^{\left(p\right)}$ is the ith neuron in the pth layer. ${W_{ij}}^{\left(p\right)}$ is the connection weight of the corresponding neuron in the current layer, $\theta_{j}$ is the threshold of the corresponding neuron, and f(x) is the activation function. The more popular activation functions now include.

(10) $$\mathrm{sigmoid}\left(x\right)=\frac{1}{1+e^{-x}}\;\mathrm{ReLU}\left(x\right)=\max\left(0,x\right)$$

The sigmoid function maps the input to [0,1] and the ReLU function maps the input to [0, x], which needs to be selected according to the needs of the application. The above formula can be written in matrix form for easy calculation.

(11) $$\vec{x^{\left(p+1\right)}}=f\left(W^{\left(p\right)}.\vec{x^{\left(p\right)}}-\theta^{\left(p\right)}\right)$$

Calculate the value of each neuron layer by layer according to the above formula to complete the forward propagation, which is also the process of classifying a new, unlabeled data input during the test.

The back propagation process is the process of modifying the model according to the difference between the forward propagation and the actual label. For this reason, we must design a reasonable optimization algorithm to make the model converge as quickly as possible and improve its performance on the training set and test set. At present, there is no theoretical hyperparameter setting method. Most training algorithms are based on the least squares optimization of the loss function E(W,θ),

(12) $$E\left(W,\theta \right)=\underset{i\epsilon D}{\sum }{\left(t_{i}-y_{i}\right)}^{2}$$

where D is the training set, $t_{i}$ is the label of the data, and $y_{i}$ is the forward propagation output.

**Figure 9:**
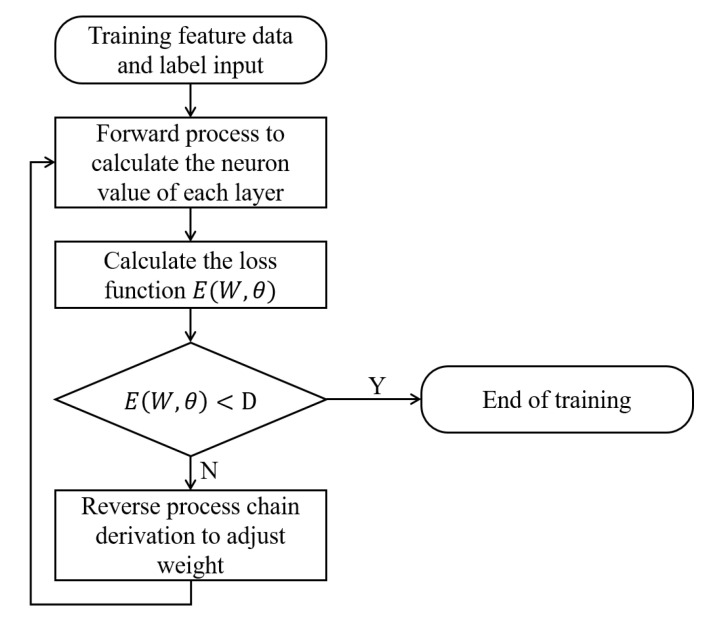
BP neural network algorithm flow chart.

The parameter update needs to calculate the partial derivative of each parameter to the loss function. Since the commonly used models generally have a larger number of parameters, to simplify the derivation process, use chain derivation:

(13) $$\frac{\partial E\left(W,\theta \right)}{\partial W}=\frac{\partial E\left(W,\theta \right)}{\partial z^{l}}\frac{\partial z^{l}}{\partial W}\;\frac{\partial E\left(W,\theta \right)}{\partial \theta }=\frac{\partial E\left(W,\theta \right)}{\partial z^{l}}\frac{\partial z^{l}}{\partial \theta }$$

where the calculation of $z^{l}=W^{\left(l\right)}.\vec{x^{\left(l\right)}}-\theta^{\left(l\right)}$, $\frac{\partial E\left(W,\theta \right)}{\partial z^{l}}$ depends on activation function form. In summary, the BP neural network can complete the classification problem in a supervised manner. It is a general method to solve the classification of large numbers of samples. It has an accuracy and flexibility that traditional clustering algorithms such as GMM and K-means cannot match and can effectively use large data samples. This improves the modeling performance, but there are also shortcomings such as slow learning speed, poor selection of hyper-parameters, local minimum, and unreasonable network structure planning. A BP neural network with five layers is used to complete target classification in proposed system. The flow chart of BP neural network algorithm used in this system is shown in Figure 9.

The advantage of BP neural network is that it can perform non-linear mapping for a large number of samples, which has a good effect on underwater acoustic data with a larger sample size. In addition, the BP neural network has self-learning and generalization capabilities, and can effectively process samples that are quite different from the training data. The disadvantage of BP neural network is that the gradient descent algorithm converges slowly to samples with higher dimensions. When the network scale is large, the convergence speed of the BP neural network is unacceptable. In addition, the training of BP network highly depends on the number of labeled samples. When the number of samples is small, it is difficult for the BP neural network to achieve better training results.

In summary, the RBM auto-encoder-BP classifier proposed in this paper mainly has the following advantages. BP neural network can effectively complete the nonlinear mapping problem and has a good effect in the clustering of RBM auto-encoders. The RBM auto-encoder can effectively compress the data and extract its abstract features, which is beneficial to speed up the convergence of the BP neural network. The generalization ability of BP neural network supplements the defect that RBM auto-encoder is difficult to effectively reconstruct untrained labels. Both BP neural network and RBM auto-encoder are self-learning algorithms, which can complete underwater acoustic target recognition tasks without manual intervention in practical applications. The data generation capability of the RBM auto-encoder can create identically distributed samples by modeling the probability distribution of the original data. When the number of samples is insufficient, the data generation capability of the RBM auto-encoder can be used to supplement the samples to complete the training of the BP neural network. Finally, RBM suppresses random noise based on the characteristics of the probability distribution of the data, which can effectively prevent the BP neural network from overfitting.

## 4. Experiment

Two noise databases were used to test the effectiveness of above-mentioned underwater acoustic target recognition system. Two comparison methods were used to verify the superiority of the proposed system in aquatic target recognition.

The experiment process was divided into three stages. First, the original audio data were preprocessed according to the needs of different algorithms, and the original audio data were divided into parameterized samples of equal length. Then, samples were randomly selected to construct training set and test set, and the number ratio was 6:1. Finally, the dataset was used to train and test the underwater acoustic target recognition system to evaluate the performance of different systems.

The number of layers of RBM auto-encoder was set to 4, and the number of units in each layer was 784, 500, 200, and 50. The learning rate during training was set to 0.001. The value of the weight matrix was initialized randomly at [−0.001, 0.001], the offset value was initialized to 0, and the number of iterations for each layer was set to 100. The Boltzmann machine took the normalized spectrum of the segmented signal as input and completed the feature extraction through the four-layer RBM auto-encoder. BP neural network obtained the output features of RBM auto-encoder to complete target recognition.

As the control group, GFCC extracted the features of audio signals after the same preprocessing. The number of gammatone filters using in GFCC was 64, and the first 24 dimensions were taken as classification features for BP neural network.

To evaluate the performance of the feature extraction algorithm in reducing the original signal’s dimensionality and improving the data separability, we set up a comparison algorithm to directly put the normalized spectrum of the original data into the BP neural network for recognition.

### 4.1. Acoustic Signal Preprocess

The data samples processed in this study are single channel audio signal. The signal was divided into 100 ms frames with 50% overlap between frames, and Hanning window was added to the signal to suppress high frequency interference and energy leakage. After adding Hanning window and FFT, spectrum samples of a frame data were obtained. The formula is as follows:

(14) $$X\left(k\right)=\left|\overset{N-1}{\underset{n=0}{\sum }}x\left(n\right)w\left(n\right)e^{-j\frac{2\pi nk}{N}}\right|\;$$

where ***N*** is the number of sampling points of a frame of data, ***w***(***n***) is the Hanning window function, and ***X***(***k***) is the norm of the spectrum.

The normalized spectrum $\hat{X}\left(k\right)$ was taken as the system input. $\hat{X}\left(k\right)$ is calculated as follows.

(15) $$\hat{X}\left(k\right)=\frac{X\left(k\right)-\min\left(X\right)}{\max\left(X\right)-\min\left(X\right)}$$

At this time, $0\leq \hat{X}\left(k\right)\leq 1$. $\hat{X}\left(k\right)$ meets the requirements of RBM for input data.

### 4.2. Experiment Result

To evaluate the performance of above-mentioned underwater acoustic target recognition system, the proposed classification system was used to carry out multiclass classification experiments on two databases, NOISEX-92 and ShipsEar. After preprocessing each signal, many samples were obtained. Six of the of the samples were randomly selected as the training set, and the remaining one was used as the test set. The training set was used to train the RBM auto-encoding BP neural network classification system, and then the test set was used to count the classification accuracy. To quantitatively describe the performance of underwater acoustic recognition system, adjusted Rand index (ARI) and Maximum Value of the Clustering Rate (MVCR) were introduced. ARI was used to evaluate the overall recognition performance. MVCR was used to evaluate the recognition performance of a single class. Assuming that the dataset of N classes is clustered into K clusters, ARI is defined as:

(16) $$\mathrm{ARI}=\frac{\sum_{ij}\left(\begin{array}{c} n_{ij}\\ 2 \end{array}\right)-\frac{2ab}{N\left(N-1\right)}}{\frac{1}{2}\left(a+b\right)-\frac{2ab}{N\left(N-1\right)}}$$

where $n_{ij}$ is defined as the number of samples that should belong to the ith clustered into the jth cluster. Higher ARI value means better clustering results.

MVCR analyzes the clustering effect of each class, which is defined as:

(17) $$\mathrm{MVCR}\left(i\right)=\frac{\max\left(n_{ij},\;j=1,2,\ldots ,K,\;j\notin \Theta_{i}\right)}{\sum_{j}n_{ij}}$$

where $\Theta_{i}$ is the set of label that has been previously selected.

The RBM auto-encoder algorithm was compared with five different clustering systems, including the two feature extraction methods GFCC and original spectrum. The clustering methods are BP neural network and GMM. The experimental results of data sets 1 and 2 are summarized in the next two sections respectively.

#### 4.2.1. Experiment Result on Dataset 1

The NOISEX-92 database was used as Dataset1 to test the performance of the classification system. NOISEX-92 is a group of noises with obvious category characteristics recorded in the air. We used NOISEX-92 to test the proposed classification system to ensure that the system can work properly. The NOISEX-92 database contains 14 types of noise. The data sample is a single channel audio signal with a duration of about 4 min, recorded with sampling rate of 19.98 kHz and bit depth of 16. The experiment used 10 types of noise in NOISEX-92. The information of each type is in Table 3. The average clustering accuracy rate, ARI, and the maximum, minimum, and average measures of MVCR were calculated by averaging 10 independent experiments. At the same time, the standard deviation of accuracy rate and ARI were calculated in 10 experiments to evaluate the robustness of the proposed classification system. The experimental results of Dataset 1 is summarized in Table 4.

#### 4.2.2. Experiment Result on Dataset 2

**Figure 10:**
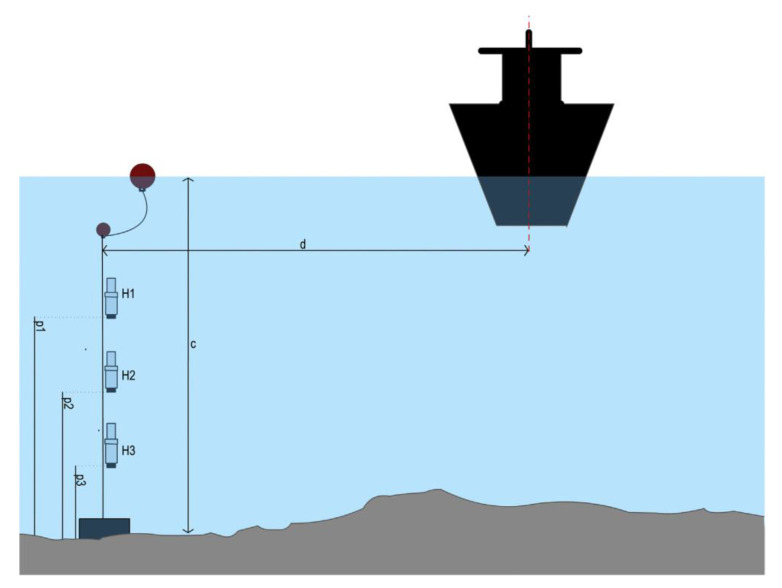
ShipsEar data collection diagram.

The ShipsEar database was used as Dataset 2 to test the performance of the proposed classification system. ShipsEar database is widely used in underwater acoustic target recognition, ship noise detection and other research fields. The database contains a large number of ship noise and marine environmental noise samples, which were recorded in autumn 2012 and summer 2013 in different parts of the Spanish Atlantic coast in northwest Spain. The schematic diagram of ShipsEar database acquisition is shown in Figure 10. The hydrophones were bottom-moored and attached to a submerged buoy to ensure verticality and a surface buoy for recovery. Hydrophones height over the bottom was selected according to water depth at the mooring point. Whenever possible, three hydrophones at different depths and with different gains were used to maximize the dynamic range of the recording. In very shallow areas (depths under 10 m), recordings were made with one or two hydrophones [32].

The amplifier used a 100 Hz high-pass filter to suppress marine background noise, the hydrophone sampling rate is 52,734 Hz, and the AD converter bit depth is 24 bits.

This experiment used all the files in the ShipsEar database. Similar to the authors of [32], we manually divided all data into five categories according to the size of the vessel. The categories are shown in Table 5. The experimental results of Dataset 2 is summarized in Table 6.

Table 4 shows that the proposed classification system on the NOISEX-92 database generally has a higher accuracy and MVCR than the other methods. It is worth noting that the proposed classification system also has better performance in terms of ARI and its standard deviation. When testing more critical actual underwater acoustic data, similar results were obtained, as shown in Table 6. Compared with the test results of Dataset 1, the performance of the method based on GFCC and original spectrum has different degrees of degradation in the test results of underwater acoustic data. The performance of the proposed method is basically consistent with that of Dataset 1, which shows that the proposed method has strong robustness under different data. The experimental result proves the effectiveness and robustness of the proposed classification system.

## 5. Conclusions

In this paper, a depth clustering algorithm combining RBM automatic encoder and BP neural network is used for underwater acoustic target recognition. This method can extract features and classify targets without manual intervention. Using a RBM auto-encoder to perform layer-by-layer self-supervised auto-encoding on the original signal can extract high-level abstract information that is beneficial to clustering. Two real acoustic databases were used to test the recognition effect of the proposed system. In two database tests, the proposed system shows better performance than the classification system based on hand-crafted features, which proves the effectiveness of the proposed system. The proposed method provides a good technical support for the target classification and recognition function of sonar system. The feature extraction and target classification of the proposed method under the condition of small sample size and low SNR is worthy of further study.

## Figures and Tables

**Table 1:** Symbol Description.

Symbol	Explanation
$x_{j}^{\left(i\right)}$	jth neurons in ith epoch
p(h|v)	Conditional probability of hidden unit under visible unit
$E_{v}\left[x\right]$	The expectation of x when the state of the visible unit is determined
$h\sim sigm\left(W^{\prime }v+c\right)$	The probability distribution of h obeys $sigm\left(W^{\prime }v+c\right)$
|x|	Modulus of complex x or vector x
$S_{i}$	Gibbs operator
ln(x)	natural logarithm of x
$\left(\begin{array}{c} n\\ m \end{array}\right)$	Number of combinations selecting m elements from n elements without repetition

**Table 2:** Detailed principle of GFCC algorithm.

Step	Process	Equation
1	Signal segmentation and windowing	$W\left(n\right)=\{\begin{array}{c} 0.54-0.46\times \cos\left(\frac{2\pi n}{N-1}\right)\;n=0,1,\ldots ,N-1\\ 0\;\;\;\;\;\;\;\;\;\;\;\;\;\;\;\;\;\;otherwise \end{array}$ $S_{w}\left(n\right)=y\left(n\right)\times w\left(n\right)$
2	Power spectrum calculation	$x\left(k\right)=\overset{N-1}{\underset{t=0}{\sum }}x\left(t\right)e^{-2\pi jtk/N},\;k\in \left[0,N\right]$ where *N* is the number of FFT points and *x*(*t*) is the input signal after windowing.
3	Gammatone filter bank parameters calculation	$g_{i}\left(k\right)=k^{n-1}e^{-2\pi B_{i}k}\cos\left(2\pi f_{i}+\varphi_{i}\right)u\left(k\right),\;i\in \left[1,Q\right]$ where n is the filter order, $B_{i}$ is the filter attenuation coefficient, $f_{i}$ is the center frequency, and Q is the number of filters in the filter bank.
4	Human hearing simulation	$\mathrm{ERB}\left(f_{i}\right)=24.7\times \left(\frac{4.37f_{i}}{1000}+1\right)$ $b_{i}=1.091ERB\left(f_{i}\right)$ where $\mathrm{ERB}\left(f_{i}\right)$ is critical frequency band of human hearing and $b_{i}$ is the bandwidth of the ith subband of the filter bank.
5	Logarithmic compression of signal energy spectrum	$Es\left(i\right)=\ln\left[\overset{N-1}{\underset{n=0}{\sum }}{\left|X\left(k\right)\right|}^{2}G_{i}\left(k\right)\right],\;i\in \left[1,Q\right]$
6	DCT transformation	$\mathrm{GFCC}\left(i\right)=\sqrt{\frac{2}{N}}\overset{Q}{\underset{j=1}{\sum }}Es\left(j\right)\cos\left[\frac{\pi i}{Q}\left(j-0.5\right)\right],i\in \left[1,Q\right]$

**Table 3:** Information of Dataset 1.

Type	Sound Level	Description
buccaneer1	109 dBA	Noise in Buccaneer
buccaneer2	116 dBA	Noise in Buccaneer
destroyerengine	101 dBA	Noise in Destroyer engine room
destroyerops	70 dBA	Noise in Destroyer operations room
f16	103 dBA	Noise in F-16 cockpit
factory1	--	Noise in Factory floor
factory2	--	Noise in Factory floor
pink	--	Pink noise
volvo	--	Noise in Volvo 340
white	--	White noise

**Table 4:** Performance of clustering system on Dataset 1.

Method	Correct ± SD	Max MVCRs	Min MVCRs	Avg MVCRs	ARI ± SD
Spectrum + GMM	65.70 ± 5.43%	**100.00%**	47.39%	67.29%	52.8 ± 5.2%
Spectrum + BP	67.20 ± 3.96%	99.93%	52.52%	70.99%	49.9 ± 3.9%
GFCC + GMM	63.91 ± 12.77%	**100.00%**	35.66%	71.35%	56.3 ± 8.5%
GFCC + BP	64.37 ± 10.39%	**100.00%**	33.16%	67.03%	55.2 ± 7.1%
RBM + GMM	82.54 ± 4.21%	**100.00%**	72.31%	83.34%	72.5 ± 6.3%
RBM + BP	**98.74 ± 0.21%**	**100.00%**	**93.34%**	**98.71%**	**97.2 ± 0.7%**

Bold data are the result of achieving the optimal performance of the test in various methods.

**Table 5:** Data classification in ShipsEar.

Category	Type of Vessel
Class A	fishing boats, trawlers, mussel boats, tugboats, and dredgers
Class B	motorboats, pilot boats and sailboats
Class C	passenger, ferries
Class D	ocean liners and ro-ro vessels
Class E	background noise recordings.

**Table 6:** Performance of clustering system on Dataset 2.

Method	Correct	Max MVCRs	Min MVCRs	Avg MVCRs	ARI
Spectrum + GMM	43.37%	87.34%	30.87%	44.12%	17.23%
Spectrum + BP	45.51%	89.92%	31.88%	46.83%	19.03%
GFCC + GMM	55.12%	90.55%	32.12%	54.42%	25.12%
GFCC + BP	54.09%	91.13%	31.09%	53.22%	24.75%
RBM + GMM	79.21%	92.31%	71.56%	82.31%	65.34%
RBM + BP	**93.17%**	**97.93%**	**89.66%**	**93.31%**	**83.91%**

Bold data are the result of achieving the optimal performance of the test in various methods.
